# Access to Hemoglobin A1c in Rural Africa: A Difficult Reality with Severe Consequences

**DOI:** 10.1155/2018/6093595

**Published:** 2018-02-26

**Authors:** Paul H. Park, Sonak D. Pastakia

**Affiliations:** ^1^Inshuti Mu Buzima/Partners In Health-Rwanda, PO Box 3432, Kigali, Rwanda; ^2^Purdue Kenya Partnership, Purdue University College of Pharmacy, PO Box 5760, Eldoret 30100, Kenya

## Abstract

Sub-Saharan Africa (SSA) continues to have the highest diabetes-related mortality rate in the world. While there exists a multitude of health system barriers driving poor diabetes control, rural facilities particularly in SSA lack access to proper monitoring of glucose and other key biologic tests. At best, most of these diabetes patients receive random blood sugar readings only on the day of their clinic visit. This approach has very limited clinical value in determining dosage adjustments for narrow therapeutic index medications such as insulin. Furthermore, access to other blood tests and physical exam tools for detecting early signs of diabetes complications is limited. We propose that routine access to hemoglobin A1c (HbA1c) testing would not only allow for close monitoring of diabetes control but also provide critical data informing the population level risk for diabetes complications. However, implementing HbA1c testing does have its own barriers at rural facilities, including high costs, refrigeration requirements, and perceived discordance between HbA1c values and mean blood glucose levels for SSA patients. Fortunately, several pilots in rural SSA have illustrated feasibility of HbA1c testing. Further political will, price reduction, and context-specific research are needed. Increasing access to HbA1c testing is a critical step to combat the high diabetes-related mortality rates in rural SSA.

## 1. Introduction

Currently, SSA has the highest diabetes-related mortality rate globally [1]. Furthermore, a higher proportion of diabetes patients in SSA develops chronic complications [2]. With the majority of patients living in rural areas, centralized diabetes provisions at referral hospitals alone are not sufficient. As diabetes is a chronic condition, expecting rural, low-income patients to consistently travel to distant referral hospitals for the remainder of their life is not practical. Unfortunately, health systems in such resource-limited settings are simply not prepared to treat DM across more remote regions [3]. Limited clinical training, screening, disease awareness among patients and clinicians, medication and diagnostic access, community-level support, and behavioral education are some of the critical challenges behind such poor patient outcomes.

With innovative foresight and political will, some countries in SSA have been able to decentralize diabetes care to the district hospitals and health centers with documented early success [4, 5]. However, in both rural and urban facilities, access to appropriate diagnostic and monitoring resources has lagged behind the growing access to medications. Unlike hypertension and other noncommunicable diseases (NCDs) which may only require physical exam and reusable medical equipment for monitoring, DM requires more technical and costly consumables. Thus, implementation gaps of well-intentioned decentralization efforts have ultimately arisen due to the high costs of essential technology and insufficient political will. Here, we describe the realities of poor diabetes monitoring capacity and its downstream consequences on morbidity and mortality at the district and health center levels for the clinicians and ultimately the patients in SSA. While there exist several well-documented essential laboratory technologies for diabetes patients including creatinine and lipids, we will specifically focus on the potential role increased access to glycosylated hemoglobin (HbA1c) may play in overcoming many of the current gaps in type 1 and 2 diabetes care. Early lessons learned surrounding community-based diabetes implementation and policy from experiences in SSA will also be shared in order to illustrate the need and impact of HbA1c testing.

## 2. The Clinical Reality of Monitoring Patients

As the majority of DM patients in rural SSA do not own a blood glucose monitoring device, the only objective monitoring of their disease typically occurs at routine clinic visits at public sector, district hospitals, or health centers [6]. A fasting blood glucose level at each visit may be informative to the clinician regarding the patient's diabetes control at the time of visit; however, this approach provides limited insight into the patient's blood glucose control between the long durations in person visits. Furthermore, patients have a tendency to strive for better glucose control in the days leading up to a clinical visit to try to impress their providers. Consequently, the clinician may never truly know the average blood sugar during the previous one to three months and is left to rely on a single blood glucose reading to guide longer-term management decisions. Even if providers assume that the patient is consistently self-managing their diabetes during the entire period between visits, the traveling distance and potential waiting times at the clinic make it unsafe to request a patient to come to the clinic in a fasting state. Thus, at each visit, the patient's diabetes control is frequently assessed by using single random blood glucose (RBG). Since RBGs have limited clinical value for outpatient monitoring, internationally validated clinical protocols for the management of DM via RBG currently do not exist. Thus, the true level of diabetes control is not known, and clinicians are then forced to make dosing changes and medication adjustments that are not based on any reliable evidence. These dynamics continue to accelerate the poor mortality patients, especially those with type 1 diabetes, in SSA face. These pragmatic dynamics often lead to very conservative medication dosing practices among providers. Because of the acute risks of hypoglycemia that could result from dose increases, providers often err on the side of minimal medication changes which could contribute to the inordinately higher blood glucose values seen among patients receiving care for diabetes in Africa [3]. By prioritizing increased access and availability to HbA1c testing, many of these trends could potentially be reversed as providers would be able to observe the average blood glucose over the antecedent three months to develop a better understanding of the patient's glycemic control. This is supported by a study from Guinea which identified access to HbA1c testing as the primary predictor of poor glycemic control [7].

There continues to exist a wide gap between the current access to monitoring being provided to patients in SSA, the minimum recommendations set forth by the International Diabetes Federation, and recommendations set forth by organizations like the American Diabetes Association (ADA) for resource-rich settings [8, 9]. While the ideal scenario would be to dramatically increase availability of laboratory tests, including HbA1c, lipids, and creatinine, to provide the full complement of diagnostic technologies recommended by organizations like the ADA, a more practical first step to help reverse the unfortunate mortality trends in SSA would be to focus on incorporating HbA1c testing into the routine care of all patients with diabetes. Prioritizing the availability of HbA1c testing provides many benefits beyond its primary purpose of being able to characterize average blood sugars over the course of 2-3 months. Through its utilization within large-scale randomized control trials like the Diabetes Control and Complications Trial (DCCT) and the United Kingdom Prospective Diabetes Study, we know that controlling HbA1c levels significantly reduces the risk of diabetes-related complications (see Table 1) [10–12]. Because of the findings from these landmark studies, HbA1c testing has the added benefit of being able to reliably predict the risk for microvascular (diabetic nephropathy, neuropathy, and retinopathy) and macrovascular complications (coronary artery disease, peripheral arterial disease, and stroke) [13]. As healthcare systems in SSA continue to mature to provide more comprehensive and reliable infrastructure for creatinine, fundoscopy, lipid testing, and arteriogram monitoring, HbA1c can serve as an interim measurement which can provide insight into the renal, ophthalmological, and cardiovascular comorbidities; these specific tests are designed to assess. Greater availability of more informative glucose control data via HbA1c testing can provide guidance to national policymakers as well as clinicians in the field regarding the risk of the wide range of diabetes-related comorbidities mentioned above. While we should continue to strive to improve access to all of these tests to improve the provision of individualized care, the introduction of HbA1c testing into care can help provide much-needed population and individual level evidence on the actual burden of diabetes and the risk of its complications.

## 3. Challenges to a Potential Solution

The primary challenge to implementing HbA1c is access. In 2012, PATH produced a report surveying DM-related resource availability in LMICs and noted that HbA1c tests were available in only 8 of 13 surveyed countries [14]. Of those with HbA1c capacity, most availed HbA1c testing via semiautomated analyzers which are located within higher-level hospitals and referral centers only. Because of this dynamic, patients residing in more remote rural areas are often required to travel long distances to receive HbA1c testing and frequently skip this testing modality entirely.

The laboratory-based HbA1c test has reliable accuracy, and each test simply requires further reagent consumption. Thus, a single test uses approximately 2.50 USD worth of reagent. However, health centers in LMICs commonly do not have stable laboratory capacity (electricity, refrigeration, trained staff, upfront machine cost and maintenance, etc.). Furthermore, the delay in turn-around time of results may prevent the clinician from being able to make timely dose adjustments on the same day of the patient visit.

Fortunately, point-of-care HbA1c technology has steadily developed to provide timely results in remote locations without requiring formal laboratory infrastructure. However, the point-of-care HbA1c technology still requires refrigeration of cartridges, which are costly and more readily available in the private sector. For example, in the Kenyan private sector, patients pay approximately 25 USD per HbA1c test compared with 10 USD in the public sector. Even with the relatively lower patient cost in the public sector, <20% of patients receive this test [15, 16]. These high costs, often times driven by local government taxes levied on imports, preclude public sector leadership from procuring the device as patients are unlikely to afford even a partially subsidized cartridge cost. As a result, the majority of rural, public health facilities continue to lack the capacity for laboratory-based or point-of-care HbA1c testing.

An additional challenge is the perceived discordance between HbA1c values and mean blood glucose levels for patients of African descent. While HbA1c measurements have become the standard of care in monitoring diabetes control for many parts of the world, its reliance on hemoglobin A is seen as a limitation for African populations given common hemoglobinopathies which may reduce the available concentration of hemoglobin A. That said, studies have shown conflicting evidence in terms of identifying a clear inaccuracy of HbA1c measurements among individuals with hemoglobinopathies [17–19]. Regardless, further studies in the form of population- level data within SSA are needed to clarify this potential for measurement bias as HbA1c availability is scaled up.

## 4. Implementation Experiences in the Public Sector in Rural SSA

Several SSA cases of HbA1c used in district and health center levels have been implemented. The International Diabetes Federation (IDF) Life for a Child program has sponsored the provision and implementation of in-kind point-of-care HbA1c testing to pediatric type 1 diabetes patients in district hospitals of 19 African countries [20]. The IDF has also supported a research grant which has brought access to 10 regional centers in Cameroon and Guinea [21]. Two further SSA HbA1c implementation projects are described below.

The Academic Model Providing Access to Healthcare (AMPATH) in western Kenya faced many of the aforementioned challenges in trying to establish diabetes services. While the initial focus was on ensuring the availability of basic medications such as insulin and a point of care glucose test at the referral hospital, we quickly realized that these efforts were life-saving but not life preserving. The subsequent introduction of HbA1c technology at our main referral center along with our rural satellite clinics emphasized this point as the average HbA1c at these clinics was 10.2% when combining the HbA1c measurements for all patients [5, 22]. Patients on insulin fared even worse with 60% of the readings for patients on insulin exceeding the maximum testing value (>14%) of our point of care device. Without the availability of HbA1c testing, we would have never realized the poor state of glycemic control of our population. This more accurate and precise awareness of glycemic control beyond simple random blood sugars helped our program improve our clinical decision making but more importantly provided the impetus for launching an early detection program [23], a home-based glucose monitoring service [24], and decentralization of services to facilitate more frequent clinical visits for patients who live in remote rural areas [5].

Since 2006, the Rwandan Ministry of Health (RMOH), with the support of Partners In Health (PIH), Rwanda, has provided chronic care for diabetes at district hospitals as part of a nurse-led integrated NCD model, which includes common NCDs, such as type 2 DM, hypertension, and asthma, as well as severe NCDs, such as type 1 DM and rheumatic heart disease [25]. Prior to 2009, these integrated NCD clinics also confronted challenges of uncertain diabetes control. Comprehensive clinical management guidelines were published; however, the implementation of protocols remained difficult without capacity to consistently monitor patients via HbA1c. Between 2006 and 2014, the median baseline HbA1c result for enrolled diabetes patients was 10.3% [4]. The team addressed this poor control with a mentored decentralized model to the health center level and improved access to HbA1c testing [26]. Among those who were tested, patients showed a significant improvement in median HbA1c from 10.3 to 8.2% after 7 to 12 months of treatment [4]. Currently, home-based glucometer and early detection programs are being explored.

## 5. Recommendations

With the many positive attributes of HbA1c testing and current limitations to access, it is clear that there must be focused efforts to urgently scale up HbA1c testing to improve the quality of diabetes care for patients in SSA. Thus, we recommend the following policy changes:

- Political will: international health entities and local governments must recognize and commit to procurement and subsidization (as necessary) of HbA1c testing as an essential laboratory test from the level of referral hospitals down to the health center level.
- Validation of HbA1c testing in SSA: population-level data is needed to further clarify potential inaccuracies of HbA1c results in SSA. As described above, both hemoglobinopathies as well as technology-related implementation challenges necessitate further validation.
- Differential pricing for public sector: manufacturers must reduce unit costs for qualified LMICs; establishing lower cost production of HbA1c would also reduce cost.
- Tax exemption: local government import taxes on HbA1c testing technology must be exempt to further drive costs down
- Innovation: create a low-cost battery-powered point of care test which eliminates the need for power, refrigeration, and expensive reagents or cartridges and can adjust for common hemoglobinopathies seen in LMIC populations.

## 6. Conclusion

The World Health Organization's Essential Medicines List (EML) has become a vehicle for producing effective advocacy for price reduction and identifying gaps in national plans. However, for diabetes, the value of including six life-saving drugs on the EML is severely hindered by the limitations in accessible diagnostics. Medicines without monitoring technology, and vice versa, have limited utility. Even if insulin were free, the clinicians would remain unable to identify the patient's level of control, and SSA will continue to have unacceptably high morbidity and mortality from a treatable illness. The same paradigm could be stated for the myriad of downstream complications, including chronic kidney disease, neuropathy, and hyperlipidemia, which all require essential diagnostic and monitoring technologies. As successful interventions illustrating the feasibility of HbA1c testing in rural SSA continue to be implemented, a clear policy-level plan for greater access to HbA1c testing must be implemented. Otherwise, the efforts of well-trained rural clinicians in SSA can only go so far, and the disheartening diabetes-related mortality rates will only be compounded by the global diabetes community's collective inaction.

## Figures and Tables

**Table 1:** Landmark studies documenting the role of HbA1c control in reducing complications.

	DCCT^∗^	UKPDS^○^
Baseline HbA1c → Final HbA1c	9.1% → 7.3%	7.9% → 7.0%
*Complications*		
Retinopathy	↓ 63%	↓ 17%–21%
Nephropathy	↓ 54%	↓ 24%–33%
Neuropathy	↓ 60%	NA
Macrovascular disease	↓ 41%^+^	16%^+^

^∗^Diabetes control and complications trial [10]. ^○^The United Kingdom prospective diabetes study [11]. ^+^Not statistically significant.
